# Arginine Metabolites as Biomarkers of Myocardial Ischaemia, Assessed with Cardiac Magnetic Resonance Imaging in Chronic Kidney Disease

**DOI:** 10.3390/biom11030416

**Published:** 2021-03-11

**Authors:** Ranjit J. Shah, Sara Tommasi, Randall Faull, Jonathan M. Gleadle, Arduino A. Mangoni, Joseph B. Selvanayagam

**Affiliations:** 1 Department of Cardiovascular Medicine, Flinders Medical Centre, Bedford Park, SA 5042, Australia; ranjitjames@gmail.com (R.J.S.); Joseph.Selva@sa.gov.au (J.B.S.); 2 Cardiac Imaging Research, South Australian Health and Medical Research Institute, Adelaide, SA 5000, Australia; 3 College of Medicine and Public Health, Flinders University, Bedford Park, SA 5042, Australia; sara.tommasi@flinders.edu.au (S.T.); jonathan.gleadle@flinders.edu.au (J.M.G.); 4 Department of Clinical Pharmacology, Flinders Medical Centre, Bedford Park, SA 5042, Australia; 5 Department of Renal Medicine, Royal Adelaide Hospital, Adelaide, SA 5000, Australia; Randall.Faull@sa.gov.au; 6 Department of Medicine, University of Adelaide, Adelaide, SA 5000, Australia; 7 Department of Renal Medicine, Flinders Medical Centre, Bedford Park, SA 5042, Australia

**Keywords:** chronic kidney disease, CKD, endothelial dysfunction, oxygen-sensitive cardiovascular magnetic resonance imaging, OS-CMR, stress T1 mapping

## Abstract

(1) Background: Cardiovascular disease (CVD) is the major cause of morbidity and mortality in patients with chronic kidney disease (CKD). Myocardial oxygenation and perfusion response to stress, using oxygen-sensitive cardiovascular magnetic resonance (OS-CMR) and stress T1 mapping respectively, are impaired in CKD patients with and without known coronary artery disease (CAD). Endothelial dysfunction, assessed by circulating levels of asymmetric dimethylarginine (ADMA) and homoarginine (HMA), promotes atherosclerosis. We hypothesized that in CKD patients, worsening endothelial dysfunction is associated with worsening myocardial oxygenation and perfusion as assessed by change in OS-CMR signal intensity (Δ OS-CMR SI) and stress T1 (ΔT1) values. (2) Methods: 38 patients with advanced CKD underwent cardiovascular magnetic resonance (CMR) scanning at 3 Tesla. OS-CMR and T1 mapping images were acquired both at rest and after adenosine stress and analyzed semi-quantitatively. Serum ADMA and HMA concentrations were assessed using mass spectrometry. (3) Results: There was no significant correlation between Δ OS-CMR SI and ADMA or HMA. Interestingly, there was a significant negative correlation seen between Δ T1 and ADMA (r = −0.419, *p* = 0.037, *n* = 30) but not between Δ T1 and HMA. (4) Conclusions: Stress T1 response is impaired in CKD patients and is independently associated with higher circulating ADMA concentrations.

## 1. Introduction

Cardiovascular disease (CVD) is common in patients with advanced chronic kidney disease (CKD) with estimates that up-to half of patients with severe CKD have significant coronary artery disease, heart failure or left ventricular hypertrophy [1,2,3]. Traditional cardiovascular risk factors such as hypertension and hypercholesterolemia, also common among advanced CKD patients, can only partly explain the increased susceptibility to CVD in this group [4].

Virtually all known cardiovascular risk factors seen in CKD patients are associated with endothelial dysfunction, a key player in the pathogenesis of CVD together with inflammation and atherosclerosis. Endothelial dysfunction is typically characterized by reduced nitric oxide (NO) synthesis, which significantly contributes to the development of atherosclerotic CVD and to deterioration in renal function [5,6,7]. The endothelium is important for the maintenance of vascular tone and homeostasis [8]. Endothelial NO synthase (eNOS) catalyzes the conversion of L-arginine (ARG) and L-homoarginine (HMA) to NO [9]. Previous studies have shown that low HMA concentrations predict adverse CVD outcomes in the CKD population [10,11,12]. Methylation of L-arginine (Arg) residues in proteins by protein methyltransferases (PMRTs) and subsequent proteolysis yields the methylated arginines L-N-monomethylarginine (LNMMA), asymmetric dimethylarginine (ADMA) and symmetric dimethylarginine (SDMA). LNMMA, ADMA and SDMA are inhibitors of eNOS activity and typically accumulate in CKD. Elevated plasma ADMA concentrations independently predict the progression of atherosclerosis, cardiovascular death and all-cause mortality [13,14,15]. SDMA does not inhibit eNOS directly but, by competing with the cationic amino acid transporter in the endothelial cell membrane, it indirectly reduces NO production by limiting intracellular arginine availability [16]. L-citrulline is a by-product when NO is synthesized from L-arginine by NOS. Arginine can also be metabolized by arginase which hydrolyses it to ornithine (ORN) and urea. Therefore, arginase may reciprocally regulate the production of NO and an increased enzyme activity can induce endothelial dysfunction [17,18,19]. While several arginine metabolites, particularly ADMA and arginine, have been investigated in relation to peripheral endothelial function, little knowledge is available regarding their role as biomarkers of myocardial ischemia in CKD and other CVD related conditions.

Myocardial oxygenation and perfusion response to stress, using oxygen-sensitive cardiovascular magnetic resonance (OS-CMR) and stress T1 mapping respectively, are impaired in CKD patients with and without known CAD [20,21]. Furthermore, the impaired OS-CMR response is associated with major adverse events (death, myocardial infarction, ventricular arrhythmia and heart failure admissions) [20].

In this study, we sought to investigate the association between several arginine metabolites and markers of myocardial ischemia in CKD patients. In particular, we hypothesized that higher concentrations of ADMA and lower concentrations of HMA are associated with worsening myocardial oxygenation and perfusion as assessed by OS-CMR and T1 responses to stress.

## 2. Materials and Methods

### 2.1. Study Population

We studied 38 patients with CKD enrolled at Flinders Medical Centre (FMC) and the South Australian Health and Medical Research Institute (SAHMRI) between 2017–2019. Patients were included if they had severe renal failure as defined by an estimated glomerular filtration rate (eGFR) < 30 mL/min/1.73 m^2^, were requiring dialysis, or had a previous renal transplant with reasonable renal function (eGFR > 45 mL/min/1.73 m^2^). Patients were excluded if they had standard MRI contraindications, asthma, second or third-degree heart block, left ventricular ejection fraction (LVEF) < 45%, or clinically overt heart failure. All participants gave written informed consent, and the study was approved by Southern Adelaide Clinical Human Research Committee (HREC/17/SAC/86).

### 2.2. Serum Biochemistry

All participants had 10 mL of blood collected for routine biochemistry, cell counts, hemoglobin, troponin T, c-reactive protein (CRP), thyroid stimulating hormone (TSH), parathormone levels (PTH), iron studies and lipid studies. eGFR was calculated from serum creatinine using the CKD-epidemiological collaboration formula.

A further sample of blood was centrifuged at 4000 rpm for 10 min at 4 °C and the serum collected and stored in a −80 °C freezer for analysis of biochemical markers of endothelial dysfunction. These samples were later thawed for analysis. Pooled plasma from 5 healthy volunteers (2 males and 3 females) were analyzed to determine the arginine metabolite values in normal individuals. For each analyte, 4 quality controls were included–unspiked pooled plasma and pooled plasma spiked with known concentrations of the analytes (low, medium and high) in order to cover all the possible ranges. Twenty μL of sample plasma, calibrator or QC were mixed with 20 μL internal standard solution (containing 1 μM d6-ADMA, 1 μM d6-SDMA, 25 μM d7-citrulline, 2 μM d4-homoarginine, and 100 μM d6-ornithine). Following the addition of 150 μL 0.1% formic acid in methanol the sample was vortex mixed for 3 min at 2000 rpm to extract the analytes. Centrifugation at 18,000× *g* for 5 min precipitated the proteins. An aliquot (5 μL) of the supernatant layer was injected into an Atlantis Hydrophilic Interaction Liquid Chromatography (HILIC) column (2.1 × 150 mm, 3 μm, Waters, Sydney, Australia) for analysis. A gradient mobile phase consisting of (A) 0.1% v/v formic acid in acetonitrile and (B) 10% v/v acetonitrile, 0.1% v/v formic acid and 10 mM ammonium formate in water was used at a flow rate of 0.4 mL/min. The starting mobile phase was 95% A, 5% B which was varied linearly over 16 min to 50 % A, 50% B then returned to the initial conditions and equilibrated for 4 min prior to injection of the next sample. The mass spectrometer was run in positive ionization mode with data collected using a Waters proprietary MS^E^ data acquisition method at low (3 V) and high collision energy ramp (8−14 V). Parent or selected fragment ions were used for detection and quantification based on their monoisotopic mass. The quadrupole Time-of-Flight (qToF) Premier (Waters, Sydney, Australia) mass spectrometer was run in positive ionization mode with data collected using Waters proprietary MS^E^ data acquisition method. Mass spectrometer settings were as follow: capillary voltage 3.0 kV, sampling cone voltage 24.0 eV, extraction cone voltage 5.0 eV, source temperature 100 °C, desolvation temperature 300 °C, cone gas flow 30 L/Hr, desolvation gas flow 400.0 L/Hr, MS^E^ function 1 collision energy 3.0 V, MS^E^ function 2 collision energy ramp 8.0–14.0 V, collision Cell Entrance 2.0, collision Exit −10.0, collision Gas Flow 0.60 mL/min. Parent or selected fragment ions were used for detection and quantitation based on their monoisotopic mass. Retention times, corresponding parent or fragment mass, MS^E^ acquisition channel and quality performance (QC) performance data (five independent determinations at each of two concentrations) for each analyte are shown in Table 1.

### 2.3. CMR Protocol

All participants underwent scanning in a 3 Tesla clinical MR scanner (Siemens, 3T Trio and Skyra). The participants were instructed to refrain from caffeine 24 h prior to the scan. All scans started with half-Fourier single-shot turbo spin echo and fast imaging with steady precession localizers. Cine images were acquired in vertical and horizontal long-axis, and 10 short-axis images covering the entire left ventricle, using a retrospective electrocardiogram (ECG) gating steady-state free precession (SSFP) sequence (repetition time (TR) 3 ms, echo time (TE) 1.5 ms, flip angle 55°, 18 phases).

For OS-CMR, a single midventricular slice was acquired at mid-diastole using a T2-prepared ECG-gated SSFP sequence (TR 2.86 ms, TE 1.43 ms, T2 preparation time 40 ms, matrix 168 × 192, Field-of-View (FoV) 340 × 340 mm, slice thickness 8 mm, Flip Angle (FA) 44°). If required, frequency scout and shim adjustments were performed to minimize off-resonance artefacts. A set of 4–6 OS-CMR images were acquired at rest during a single breath-hold over six heart beats. Six stress OS-CMR images identical to the ones acquired at rest were acquired at peak adenosine stress (140 μg/kg per min) 90 s after initiation for at least 3 min. Stress heart rate and blood pressure were measured at 1 min intervals during adenosine infusion. Each participant was questioned about the occurrence of side effects during adenosine administration, e.g., chest pain or tightness, shortness of breath, flushing, headache, and nausea.

Shortened Modified Look-Locker Inversion Recovery (ShMOLLI) T1-maps were based on 5–7 images with specific Inversion Time (TI)~100–5000 ms, collected using SSFP readouts in a single breath-hold, typically: TR/TE~201.32/1.07 ms, flip angle = 35°, matrix = 192 × 144, 107 phase encoding steps, interpolated voxel size = 0.9 × 0.9 × 8 mm, cardiac delay time TD = 500 ms; 206 ms acquisition time for single image. Native T1 mapping were performed on 3 slices (basal, mid-ventricular and apical) at mid-diastole with a complete rim of myocardium (basal slice) and LV cavity visible (apical slice). During adenosine stress (140 μg/kg per minute), another set of 3 T1 maps were acquired on slices corresponding to the rest images using the same protocol. The images were acquired, while continuing adenosine stress, every 40 s starting at 250 s after initiation. 

### 2.4. CMR Analysis

CMR analysis was performed with CMR^42^ Version 4.1–5.9 (Circle Cardiovascular Imaging Inc, Calgary, Canada). Left ventricular mass and left and right ventricular volumes and functions were calculated using the 3-dimensional short-axis stack by tracing the endocardial and epicardial contours in end-diastole and end-systole. Left ventricular mass, left and right ventricular end-diastolic volumes, and end-systolic volumes were indexed to body surface area. 

Analysis of OS-CMR SI was performed as previously described [22]. The CMR software measured Myocardial SI after manually tracing the endocardial and epicardial contours. Each midventricular short-axis OS-CMR image was divided into 6 segments (anterior, anterolateral, inferolateral, inferior, inferoseptal, and anteroseptal) according to the American Heart Association 17-segment model. The mean myocardial SI within each segment was obtained, both at rest and stress, and corrected for variations in heart rate with the following equation previously described [22]:

SI = SI_0_/(1 − βe^−TR/T1^) (1)

where, T1 = 1220 ms and β = 0.59 (determined empirically for this sequence), SI_0_ = the measured signal intensity, SI = signal intensity corrected to heart rate, and TR is the image-dependent time between acquisition of sections of k-space, governed by the heart rate (replaced by the RR interval). The SI change was calculated as:

ΔSI = (SI_Stress_ − SI_Rest_)/SI_Rest_ × 100 (2)

Myocardial T1 analysis was performed on the T1 maps acquired during rest and stress. The CMR software measured Myocardial T1 after manually tracing the endocardial and epicardial contours. The 3 short-axis T1 map images (basal, mid-ventricular and apical) were divided into 16 segments according to the American Heart Association 17-segment model. The mean myocardial T1 within each segment was obtained, both at rest and during stress. The T1 change was then calculated as:

ΔT1 = (T1_Stress_ − T1_Rest_)/T1_Rest_ × 100%

### 2.5. Statistical Analysis

All analysis was performed using SPSS software package (IBM Corporation, Armonk, NY, USA). Descriptive statistics are presented as mean ± standard deviation for normally distributed continuous variables, median (inter-quartile range) for skewed continuous variables and as frequency (%) for categorical variables. The analyzed associations between clinical and demographic variables and individual arginine metabolites using either simple or partial correlation analysis. A *p*-value <0.05 was considered statistically significant.

## 3. Results

### 3.1. Baseline Characteristics

Table 2 details the baseline characteristics of the 38 CKD patients. The average age was 62.2 ± 13.5 years and 63% were male. Twenty patients had diabetes mellitus, 16 were on dialysis and 12 had dyslipidemia. Twelve patients had diagnosed coronary artery disease (CAD) of whom 6 had undergone revascularization (5 had coronary artery bypass grafting and 1 had percutaneous intervention with a stent inserted).

### 3.2. Arginine Metabolite Concentrations in Normal Volunteers and CKD Patients 

Table 3 shows the comparison between arginine metabolite concentrations in normal volunteers and CKD patients. As expected, CKD patients had significantly higher ADMA (0.780 ± 0.149 µM vs. 0.505 ± 0.019 µM) and SDMA (1.784 ± 0.669 µM vs. 0.505 ± 0.037 µM) concentrations and lower HMA (1.28 ± 0.61 µM vs. 2.40 ± 0.14) concentrations when compared to controls.

### 3.3. Arginine Metabolite Concentrations in CKD Patients with and without CAD

**Figure 1:**
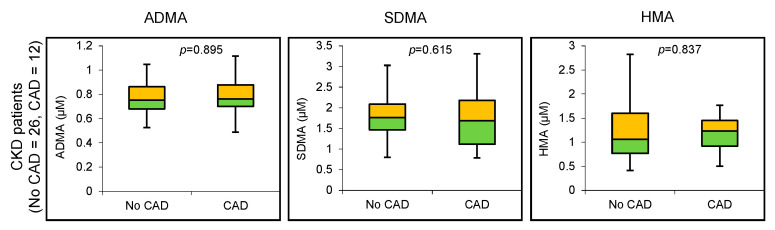
Arginine metabolite levels in CKD patients with and without coronary artery disease (CAD).

There were no significant differences in the concentrations of ADMA, SDMA or HMA between CKD patients with and without CAD (Figure 1).

### 3.4. Association between Arginine Metabolites and Patient Baseline Characteristics: Age, Gender, BMI, Renal Function (eGFR), Diabetes Mellitus, Hypertension, Dialysis, Troponin T (TnT) and hs-CRP

**Figure 2:**
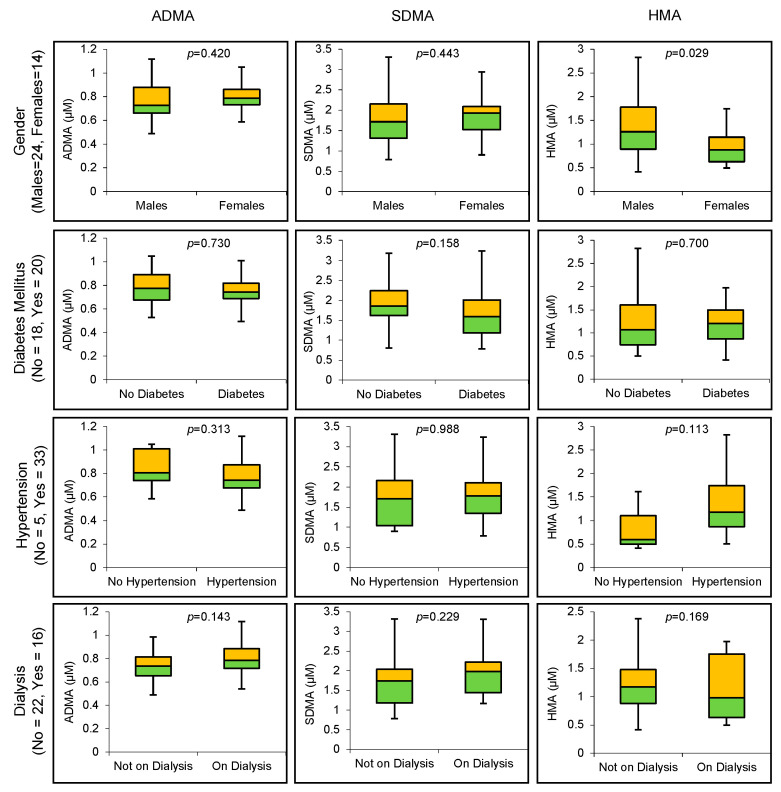
Association between arginine metabolites and patient baseline characteristics: gender, diabetes mellitus, hypertension and dialysis.

HMA concentrations were significantly higher in male patients (*n* = 24) compared to female patients (*n* = 14) with CKD (1.376 ± 0.610 µM vs. 0.962 ± 0.396 µM, *p* = 0.029). There was a trend for HMA to be higher in hypertensive patients (*n* = 5) compared to non-hypertensive patients (*n* = 33) with CKD (1.281 ± 0.566 vs. 0.845 µM ± 0.507 µM, *p* = 0.113). By contrast, gender, diabetes mellitus, hypertension or having dialysis did not significantly influence the concentrations of arginine metabolites (Figure 2).

**Figure 3:**
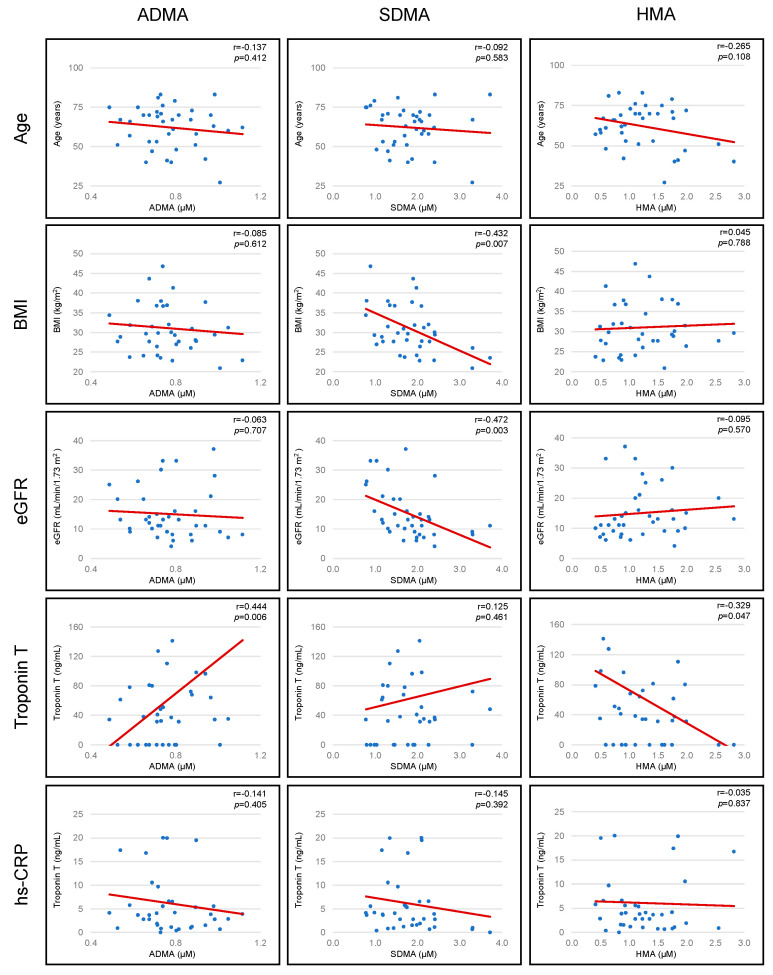
Association between arginine metabolites and patient baseline characteristics: Age, BMI, eGFR, troponin T and hs-CRP.

SDMA concentrations were inversely associated with BMI (r = −0.432, *p* = 0.007). Worsening renal function (as denoted by reducing eGFR) was associated with higher SDMA concentrations (r = −0.472, *p* = 0.003). Troponin T concentrations were positively correlated with ADMA concentrations (r = 0.444, *p* = 0.006) and negatively correlated with HMA concentrations (r = −0.329, *p* = 0.047). There was a trend for HMA concentrations to fall with increasing age (r = −0.265, *p* = 0.108). No further associations were observed between age, BMI, eGFR, Troponin T, and high sensitivity c-reactive protein (hs-CRP) and arginine metabolites (Figure 3).

### 3.5. Association between Arginine Metabolites and CMR Derived Parameters: Left Ventricular Ejection Fraction (LVEF), Indexed Left Ventricular Mass (LVMi), Stress OS-CMR and Stress T1 Mapping

**Figure 4:**
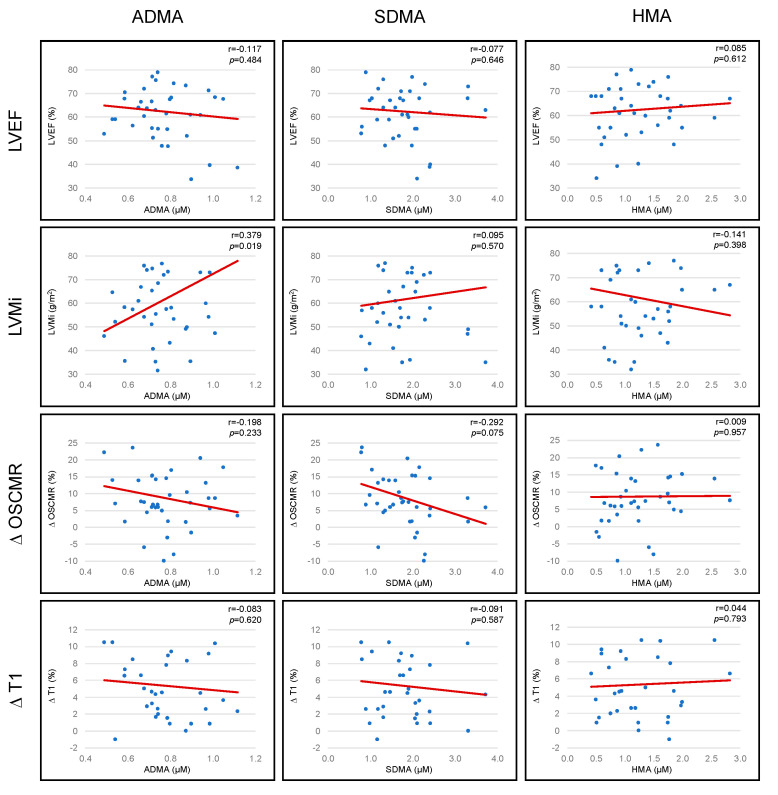
Association between arginine metabolites and cardiovascular magnetic resonance (CMR) derived parameters: LVEF, LVMi, stress OSCMR and stress T1.

ADMA concentrations were significantly and positively associated with indexed left ventricular mass (r = 0.379, *p* = 0.019). There was also a trend towards an inverse association between SDMA concentrations and Δ OS-CMR SI (r = −0.292, *p* = 0.075). No further associations were observed between LVEF, LVMi, stress OSCMR and stress T1 mapping and arginine metabolites (Figure 4).

### 3.6. Adjusted Partial Correlations between Biochemical Markers of Endothelial Dysfunction (Arginine Metabolites) and Myocardial Oxygenation (Δ OS-CMR SI) and Perfusion Response to Stress (Δ T1)

Table 4 shows the partial correlation values between arginine metabolites and OS-CMR and T1 mapping after adjusting for age, gender, body mass index, C-reactive protein and troponin T. There was no significant correlation between Δ OS-CMR SI and any of the metabolites. However, there were significant negative correlations between Δ T1 and ADMA (r = −0.419, *p* = 0.037, *n* = 30), L-citrulline (r = −0.444, *p* = 0.026, *n* = 30) and ornithine (r = −0.460, *p* = 0.021, *n* = 30). There was a negative trend for the adjusted partial correlation between Δ T1 and L-arginine (r = −0.338, *p* = 0.098, *n* = 30) but no correlation between Δ T1 and SDMA, HMA or L-NMMA.

## 4. Discussion

This is the first study to assess the relationship between biochemical markers of endothelial dysfunction and novel gadolinium-free CMR methods of assessment of myocardial ischaemia in CKD patients. In particular, there was a significant negative association between Δ T1 response and ADMA and Ornithine concentrations. In contrast, Δ OS-CMR SI was not significantly associated with arginine metabolites.

In CKD, endothelial dysfunction and atherosclerosis are almost universal, and cardiovascular complications are highly prevalent. Endothelial cell damage or injury is invariably associated with such clinical conditions as thrombosis, hypertension, renal failure and atherosclerosis and may also be responsible for accelerated atherosclerosis in patients with CKD. Although traditional risk factors are common among CKD patients, they can only in part explain the increased susceptibility to CVD [4]. Non-traditional cardiovascular risk factors (like hypophosphatemia, anemia, chronic inflammation) are important in the pathogenesis of CVD in CKD [23,24,25]. These non-traditional risk factors result in endothelial dysfunction which has a central role in the pathogenesis of CVD. Considering that CKD patients are more likely to die of CVD than to progress to end-stage renal disease (ESRD), the search for novel CVD risk factors in this population may yield novel therapeutic targets.

In this study, myocardial perfusion response to stress (as denoted by the Δ T1 response), but not myocardial oxygenation response to stress (as denoted by stress Δ OS-CMR SI response) was associated with biochemical markers of endothelial dysfunction, particularly ADMA, in the CKD population. The presence of diffuse interstitial fibrosis in CKD patients would have resulted in higher resting T1 values [26,27] and concomitant lower Δ T1 values. In contrast, OS-CMR represents tissue level oxygenation, and is represented by both blood flow and myocardial oxygenation [28]. In CKD, there is a chronic reduction in myocardial blood flow due to epicardial and microvascular coronary artery disease. Therefore, the myocardium in CKD may behave in a similar manner to hibernating myocardium which is characterized by chronic reductions in flow and function that occur in the absence of infarction or subjective evidence of acute ischemia. In normal hearts, acute transient ischemia causes dysfunction that is accompanied by a rapid fall in high-energy phosphate levels and a switch to anaerobic glycolysis as evidenced by regional lactate production. The attenuation in function of hibernating myocardium represents an adaptive state where function and metabolism are downregulated and thus oxygen consumption is reduced to limit the development of metabolic ischemia (as demonstrated by lactate production) in response to stress [29]. Thus, while blood flow is reduced at rest and the difference in perfusion between normal and hibernating myocardium increases during stress, it is not truly an ischemic state. This may be an explanation to our finding that CMR markers of perfusion (stress T1 mapping) correlated with biochemical markers of endothelial dysfunction whereas the CMR marker of myocardial ischaemia (stress OS-CMR) did not correlate with biochemical markers of endothelial dysfunction despite the fact that endothelial dysfunction and myocardial ischaemia are common in CKD patients.

Another important finding in this study was that ADMA and SDMA concentrations were significantly higher, and HMA concentrations significantly lower, in patients with CKD when compared to the normal volunteers. This can be regarded as indirect evidence of endothelial dysfunction in the CKD cohort, and in line with previous studies where patients with mild-moderate CKD have elevated plasma levels of ADMA and SDMA compared to normal controls [30,31,32,33]. This increase is more pronounced in ESKD [34,35].

Endothelial function (as indirectly assessed by measuring levels of ADMA and HMA) was noted to be worse in females, in older age and in those with increased LVMi and troponin T levels. Furthermore, ADMA, SDMA and HMA were not significantly different in the CKD patients with CAD when compared to CKD patients without CAD. Similar to CAD, CKD is associated with higher concentrations of ADMA and SDMA and lower concentrations of HMA. This might have diluted the difference between the 2 groups (CKD without CAD versus CKD with CAD).

### Limitations

Due to the relatively small sample size, these results are hypothesis-generating and therefore need to be confirmed with larger studies. Secondly, CTCA or invasive coronary angiogram was not performed (due to advanced renal failure) in this patient cohort and therefore it may be possible that some patients might have had significant undiagnosed CAD. However, care was taken while recruitment to exclude patients with known CAD or even symptoms suggestive of CAD. Thirdly, endothelial function was not directly assessed with invasive or non-invasive methods. Lastly, although image quality was generally good, artefacts could not always be resolved with frequency and shim adjustments and this could lead to reduced specificity.

## 5. Conclusions

This study demonstrates that there is a significant association between ADMA concentrations and Stress T1 response in CKD patients. However, Δ OS-CMR SI was not significantly associated with circulating ADMA or HMA.

These results need to be confirmed with larger, multi-centre studies to investigate the prognostic role of ADMA in CKD patients with CMR-documented CAD. Further studies may allow also inclusion of patients with less severe kidney disease.

## Figures and Tables

**Table 1:** Retention times, corresponding parent or fragment mass, MS^E^ acquisition channel and quality performance (QC) performance data (five independent determinations at each of two concentrations) for each analyte.

Analyte	RT (min)	MS^E^ Channel	Ion	*m/z* ^1^	Int Std	QC_low_^2^ %CV	QC_high_^2^ %CV
ADMA	11.93	1	Parent	203.16	d6-ADMA	6.4	7.1
L-ARG	11.06	1	Parent	175.13	d4-HMA	9.0	10.0
CIT	9.34	1	Fragment	159.09	d7-CIT	6.1	4.0
HMA	11.15	1	Parent	189.15	d4-HMA	1.6	1.4
L-NMMA	11.38	1	Parent	189.15	d4-HMA	8.6	14.4
ORNITHINE	11.38	1	Parent	133.11	d6-ORN	5.1	3.5
SDMA	11.75	1	Parent	203.16	d6-SDMA	4.2	4.3

Legend: ADMA, asymmetric dimethylarginine; L-ARG, L-arginine; CIT, L-citrulline; HMA, L-homoarginine; L-NMMA, N^G^, monomethyl-L-arginine; ORN, L-ornithine; SDMA, symmetric dimethylarginine. ^1^ *m*/*z* for the positively charged analyte ion, that is [M+H]^+^, data was extracted with a mass window of 0.05 Da; ^2^ QC_low_ and QC_high_ were prepared by spiking pooled human plasma (*n* = 5) with a known amount of analyte.

**Table 2:** Baseline Characteristics.

	CKD Patients (*n* = 38)
Age (years)	62.2 ± 13.5
Male sex	24 (63.2)
BMI (kg/m^2^)	31.1 ± 7.4
eGFR (mL/min/1.73 m^2^)	15.1 ± 8.5
Dialysis	16 (42.1)
Diabetes Mellitus	20 (52.6)
LVEF (%)	62.3 ± 11.7
LVMi (g/m^2^)	61.7 ± 18.6
Dyslipidemia	12 (31.6)
Smoking History	8 (21.1)
Anti-platelet Agent	15 (39.5)
Beta blocker	16 (42.1)
ACE inhibitor	10 (26.3)
Angiotensin Receptor Blocker	10 (26.3)
Calcium channel blocker	19 (50.0)
Statin	23 (60.5)

Data are presented as *n* (%) or mean ± SD. Legend: BMI, body mass index; eGFR, estimated glomerular filtration rate; ACE, angiotensin-converting enzyme; LVEF, left ventricular ejection fraction; LVMi, left ventricular mass indexed to body surface.

**Table 3:** Arginine metabolites in chronic kidney disease (CKD) patients and in normal volunteers.

Analyte	Normal Volunteers (Pooled Plasma of 5 Healthy Volunteers)	CKD Patients (*n* = 38)	*p*-Value ^1^
ADMA (µM)	0.505	0.780 ± 0.149	<0.001
L-ARG (µM)	213.8	182.7 ± 39.6	<0.001
CIT (µM)	42.6	96.7 ± 29.0	<0.001
HMA (µM)	2.40	1.28 ± 0.61	<0.001
L-NMMA (µM)	0.115	0.101 ± 0.041	0.042
ORN (µM)	71.1	84.7 ± 22.5	0.001
SDMA (µM)	0.505	1.784 ± 0.669	<0.001

Data are presented as *n* (%) or mean ± SD. Legend: ADMA, asymmetric dimethylarginine; L-ARG, L-arginine; CIT, L-citrulline; HMA, L-homoarginine; L-NMMA, N^G^, monomethyl-L-arginine; ORN, L-ornithine; SDMA, symmetric dimethylarginine. ^1^ Using one-sample *t*-test.

**Table 4:** Partial correlation values between the arginine metabolites and OS-CMR and T1 mapping after adjusting for age, gender, body mass index, C-reactive protein and troponin T.

Analyte	OS-CMR (*n* = 38)		T1 Mapping (*n* = 30)	
	*r*-Value	*p*-Value	*r*-Value	*p*-Value
ADMA (µM)	−0.106	0.558	−0.419	0.037
L-ARG (µM)	−0.183	0.308	−0.338	0.098
CIT (µM)	−0.102	0.573	−0.444	0.026
HMA (µM)	−0.164	0.362	0.000	0.999
L-NMMA (µM)	0.078	0.666	−0.002	0.992
ORN (µM)	−0.156	0.387	−0.460	0.021
SDMA (µM)	−0.226	0.206	−0.080	0.702

Legend: ADMA, asymmetric dimethylarginine; L-ARG, L-arginine; CIT, L-citrulline; HMA, L-homoarginine; L-NMMA, N^G^, monomethyl-L-arginine; ORN, L-ornithine; SDMA, symmetric dimethylarginine.

## Data Availability

The data presented in this study are anonymized and stored appropriately. This data can be made available on request from the corresponding author.
